# Joining Characteristics of 60-Layered Cu Foil Stack Using Linear Vibration Ultrasonic Welding

**DOI:** 10.3390/ma19040782

**Published:** 2026-02-18

**Authors:** Seong Min Hong, Bum-Su Go, Hee-Seon Bang

**Affiliations:** 1 Joining and Welding Research Institute, The University of Osaka, Osaka 567-0047, Japan; hong.sm.jwri@osaka-u.ac.jp; 2 Research Institute, Korea Welding and Joining Engineering Association, Gwangju 61052, Republic of Korea; 3 Department of Welding and Joining Science Engineering, Chosun University, Gwangju 61452, Republic of Korea

**Keywords:** Li-ion battery, ultrasonic welding, 60-layered Cu foil stack, welding energy, joint strength

## Abstract

This study investigates the joint characteristics of a 60-layered copper foil stack using linear vibration ultrasonic welding for lithium-ion pouch cell applications. With increasing demand for high-capacity electric vehicle batteries, ensuring the reliability of multilayer electrode joints is essential. Experiments were conducted by varying vibrational amplitude, welding time, and clamping pressure. Weld quality was analyzed based on indentation profiles, joint strength, and failure modes. Results revealed that optimal welding energy (500–900 J) produced well-formed joints without surface cracks or tearing. Excessive welding energy (>900 J) led to material thinning and interfacial failure. The maximum T-peel peak load of 138.7 N was obtained at the 30th joining interface under 25 µm amplitude, 0.8 s welding time, and 1.5 bar clamping pressure. Interface-dependent optimum conditions were observed, reflecting thickness–direction variations in deformation and bonding within the 60-layer stack. Indentation length and depth correlated linearly with welding energy. Failure modes transitioned from no adhesion to tearing and button-pull types. The findings provide guidelines for optimizing welding parameters for high-quality multilayer foil joints in battery manufacturing.

## 1. Introduction

With the growing demand for extended driving ranges in electric vehicles, multilayering and thinning of electrode foils in pouch cells have become essential strategies to achieve the required energy density and capacity. Since the battery cell electrodes are electrically connected, the quality of welded joints is a key factor in determining overall battery performance. Among the various joining methods, ultrasonic welding—a solid-state process utilizing high-frequency (20 kHz) vibrational energy—is widely used for tab welding in multilayer battery foil stacks due to its rapid cycle time, low thermal input, and compatibility with dissimilar materials.

However, the inherent characteristics of ultrasonic welding—namely, intense shear deformation and localized frictional heat—can induce critical defects such as interfacial gaps, excessive thinning, and tearing, especially when applied to ultra-thin copper foils below 10 µm. These materials are particularly vulnerable to damage caused by oscillation-induced plastic deformation [1].

Previous studies have investigated the challenges of ultrasonic welding in multilayer foil configurations. Kim et al. [1] and Kumar et al. [2] emphasized the importance of precise process control to mitigate delamination and ensure stable bonding in lithium-ion battery manufacturing. Shen et al. [3] used 3D finite element modeling to analyze the dynamic behavior of materials during welding and identified the influence of tool geometry and vibration parameters. Chen and Zhang [4] showed that the knurl pattern on the sonotrode plays a critical role in nugget formation, while Go et al. [5] explored optimization strategies for dissimilar Al/Cu joints. Recently, Choi et al. [6] introduced machine learning algorithms for real-time weld quality prediction in multilayer Cu foil welding. Recent investigations have further advanced the understanding of bonding mechanisms in multilayer copper foil welding for battery applications. Kulkarni et al. [7] systematically examined the bonding mechanisms in ultrasonic-welded multilayered copper foils for electric vehicle battery cells, revealing the critical role of interfacial friction and material flow in achieving robust joints. Complementing this, Kwon et al. [8] developed a thermo-mechanical coupled finite element model specifically for multilayer copper foil stacks, demonstrating that temperature distribution and stress concentration patterns vary significantly across layer positions. Zhao et al. [9] conducted orthogonal experiments on multilayer battery foils and tabs, identifying optimal parameter combinations through statistical analysis and bonding quality assessment. These studies collectively highlight the complexity of multilayer welding and the need for layer-specific process optimization.

Comparative studies between ultrasonic and alternative welding technologies have provided valuable insights for battery manufacturing. Kumar et al. [2] compared laser and ultrasonic welding for pouch cell multi-foil current collectors, concluding that ultrasonic welding offers superior joint consistency and lower thermal damage for thin foil stacks. Parameter optimization has been addressed through various methodologies: Yao et al. [10] applied response surface methodology to optimize ultrasonic welding parameters for battery tabs, establishing predictive models for weld quality, while Goodarzi et al. [11] employed design of experiments to optimize Al–Cu dissimilar joints, demonstrating the importance of systematic parameter selection in achieving defect-free welds.

Understanding microstructural evolution and failure mechanisms is essential for ensuring joint reliability. Cheng et al. [12] investigated the microstructural evolution in ultrasonic welding of copper cables, revealing the relationship between grain refinement, porosity formation, and tensile strength. Das et al. [13] explored the effects of Ni coating on Al/Cu ultrasonic joints, demonstrating that interface modification can significantly enhance joint performance and reduce intermetallic brittleness. Deng et al. [14] examined failure behaviors in Al/Cu joints with various interlayers (Ni, Zn, Mg), identifying distinct failure modes associated with different interlayer materials and providing guidance for material selection in dissimilar metal joining.

Recent comprehensive reviews and advanced modeling approaches have synthesized current knowledge and pointed toward future research directions. Zhao et al. [15] provided an extensive review of ultrasonic welding of copper alloys, covering process fundamentals, joint formation mechanisms, and industrial applications across various sectors including battery manufacturing. Bahavar and Karafi [16] presented numerical analysis and performance evaluation of ultrasonic metal welding machines, offering insights into equipment design optimization and process capability enhancement. These comprehensive works underscore the maturation of ultrasonic welding technology while identifying remaining challenges in high-layer-count applications.

Despite these advances, few experimental studies have addressed foil stacks exceeding 50 layers. Consequently, the joint formation mechanisms, weldability, and failure behavior of high-layer-count stacks remain poorly understood. In particular, there is a lack of systematic investigation into how indentation morphology, nugget development, and interfacial bonding are influenced by key parameters such as amplitude, welding time, clamping pressure and energy input.

Commercial pouch-cell current collectors typically require the joining of tens of thin foils, and the number of layers is increasing with the demand for higher capacity and energy density. In pouch-cell manufacturing, current collectors often require the joining of tens of thin foils, and the layer count is increasing as higher-capacity designs demand more stacked electrodes. Motivated by this trend, we selected a 60-layer Cu foil stack to represent a high-layer-count configuration and to investigate weldability and quality limits beyond the commonly studied lower-layer stacks. While prior ultrasonic welding studies have mainly focused on lower-layer stacks or foil-to-tab configurations, systematic evidence for stacks exceeding 50 layers remains limited. Here, we study a 60-layer (8 µm) Cu foil stack and provide: (i) quantitative scaling of indentation length/depth with machine-reported welding energy, (ii) an energy-based process window that minimizes interfacial gaps while avoiding thinning/tearing, and (iii) interface-resolved T-peel responses (15th/30th/45th) linked to failure mode transitions. These results offer practical quality guidelines for high-layer-count foil stack joining in battery manufacturing.

## 2. Research Methods

### 2.1. Used Materials and Process Parameters

**Figure 1:**
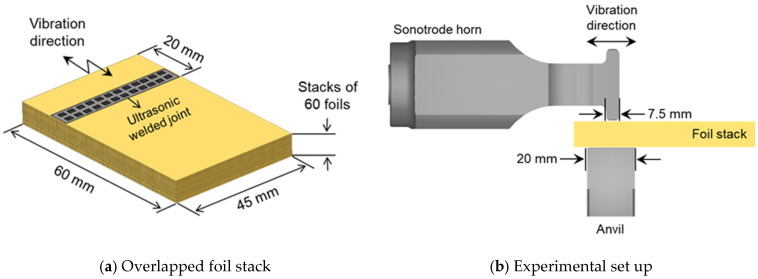
Schematic illustration of foil stack specimen for ultrasonic welding; (**a**) overlapped foil stack, (**b**) experimental set up for ultrasonic welding.

In this study, 60 layers of 8 µm-thick battery-grade bare copper (Cu-PHC) foil were selected as anode current collectors. As shown in Figure 1, the copper foils were machined as a coupon shape specimen with dimension of 60 mm (L) × 45 mm (W), and the joining configuration was completed by overlapping as a foil stack. To ensure consistent foil alignment during stacking, the 60 foils were assembled using a dedicated stacking fixture/jig that constrains the reference edges. After stacking, the foil pack was lightly pre-clamped to minimize lateral sliding during tool approach, and specimens with visible edge offset were discarded prior to welding. The chemical compositions and mechanical properties of Cu foil are listed in Table 1 [2]. Note: “Bal.” denotes the balance (remainder) of the composition. “Elongation (%)” refers to the total engineering elongation to fracture obtained from a uniaxial tensile test.

The ultrasonic welding was performed by Herrmann Ultraschall ECM 20 ultrasonic welding system (generator output: 4800 W; Herrmann Ultraschalltechnik GmbH & Co. KG, Karlsbad, Germany) with a vibration frequency of 20 kHz. The ultrasonic horn was made of heat-treated high-speed steel (HSS) with a rectangular shape surface area of 352.5 mm^2^ (47 mm × 7.5 mm), while the anvil was made of tool steel with a rectangular shape surface area of 1200 mm^2^ (60 mm × 20 mm). The surfaces of sonotrode horn and anvil were designed by a squeeze-type pattern with quadrangular pyramids. These quadrangular pyramids, called knurls, were machined mechanically to prevent damage to the base material. Each welding condition was repeated for *n* = 5 independent specimens prepared from separately stacked foil packs. For all conditions, indentation measurements and T-peel peak loads are reported as mean ± standard deviation. To reduce handling-induced variability, specimen preparation and measurements were performed following an identical procedure.

In order to verify the influence of process parameters on weldability of foil stack, the experiments were carried out under the welding conditions as shown in Table 2, and a time control mode was used. The welding energies ranged from 110 to 1852 J and corresponded with welding conditions, which were adjusted automatically from the controller. In the present study, clamping pressure, vibrational amplitude and welding time have been divided into 3, 3 and 4 levels, respectively. Moreover, joint quality was classified in terms of welding energy. The experiment was performed on a total of 36 welding conditions, with 5 replicates for each condition.

### 2.2. Joint Appearance, Mechanical and Metallurgical Characteristics

After ultrasonic welding, joint profiles were examined on both the ultrasonic horn side and the anvil side. The indentation geometry on the horn side was quantified at the weld center using an optical stereomicroscope (SZ61; Olympus Corporation, Tokyo, Japan) with an image analysis program (i-Solution, version M15.1; Image and Microscope Technology Inc., Vancouver, BC, Canada), where the indentation length (D1, parallel to the vibration direction) and width (D2, transverse to the vibration direction) were measured. In addition, visual inspection was conducted to identify macroscopic defects such as surface cracks and tearing.

To evaluate the mechanical performance of the welded foil stack joint, a T-peel (peel-dominated opening) test was employed. This configuration was selected because the multilayer Cu foils are highly compliant and thin, and thus tensile–shear testing can be significantly influenced by specimen bending, grip misalignment, and premature tearing, which may obscure the interfacial bonding integrity. In contrast, the T-peel test promotes interfacial crack opening and propagation, providing high sensitivity to partial bonding, interfacial gaps, and layer-by-layer delamination within the stack. The joint strength was defined as the peak load in the load–displacement curve, and the failure energy was calculated as the area under the load–displacement curve up to final separation. Results are reported as the mean ± standard deviation of *n* = 5 specimens for each condition.

Prior to testing, the two arms of the specimen were bent to 90° using a controlled bending procedure, with the bend line located sufficiently away from the welded zone and a defined bending radius to minimize the risk of introducing pre-cracks at the joint; after bending, specimens were visually inspected to confirm the absence of visible cracks/tears near the weld. The joining interfaces within the foil stack were designated by the number of layers from the horn side as the 15th (15 layers), 30th (30 layers), and 45th (45 layers) joining interfaces. During the T-peel test, an initial grip separation of 40 mm was used to avoid interference with specimen deformation during clamping.

All the specimens for metallurgical observation were polished using silicon carbide sandpapers from 2000 to 4000 grit and diamond suspension of 3 and 1 µm. After polishing, the metallographic specimens were etched with solution of 100 mL Ethanol, 6 mL HCL and 20 g FeCl_3_ for 10 s in sequence to observe the microstructure using optical microscope (BX51M; Olympus Corporation, Hachioji-shi, Tokyo, Japan) with an image analysis program (i-Solution, version M15.1; Image & Microscope Technology Inc., Vancouver, BC, Canada).

In this study, “welding energy” refers to the machine-reported energy integrated over the welding duration (Ew = ∫P(t)dt), and it is used as a process-integrated descriptor reflecting the combined effects of welding time, vibrational amplitude, and clamping pressure.

## 3. Results and Discussion

### 3.1. Joint Profiles

Table 3, Table 4 and Table 5 summarize representative joint profiles obtained from one-factor sweeps (welding time, vibrational amplitude, or clamping pressure) while keeping the remaining settings constant. Indentation depth values are reported as mean ± SD from five repeated welds for each condition (*n* = 5). Because the machine-reported welding energy is an outcome variable that inherently changes with any of these settings, each condition is reported together with its corresponding welding energy. The purpose of Section 3.1 is therefore to document how the surface indentation and cross-sectional features evolve under practically used parameter changes, and to provide the profile-level evidence that is later consolidated on a common basis using welding energy in Section 3.2 and Section 3.3.

On the surface of foil stack on the sonotrode horn side, it can be observed that the indentations were formed depending on the pattern of the knurl, indicating that the initial interfacial joining and welding nugget developed in this area. The area density of indentations tends to increase with the increase in welding energy. Meanwhile, on the surfaces excluding the indentations, the material compaction occurs in the area corresponding to the contact with the surface of sonotrode horn. This is due to the material property that copper foils easily deflect and deform for relatively low loads. As the welding energy increases, cracks and tearing occur on the upper and lower edges of joint surface. On the other hand, the wrinkles and bulging shape appeared around the joint surface due to shear deformation caused by ultrasonic oscillation.

In the case of the indentation of the anvil side on the surface, the pattern of indentations appeared irregular; as welding energy increases, there is a tendency to increase for the size of the indentations. Especially, at the alignment of the sonotrode horn and anvil, the size of the indentations increased significantly at the points where both knurls of the edges came into contact. From the results it was found that welding time of 0.4 s, material compaction and indentation gradually began to develop. However, at a welding time of 0.8 s cracks appeared due to excessive welding energy.

The macro-graph and indentation depth were observed to quantitatively investigate the welding defects and indentation depth of the foil stack joint. It should be noted that sectioning, mounting, and polishing could affect the shape of the indentation, cracks, and tears on the cross-section due to the ultra-thin foil.

Table 4 shows the cross-sectional view of foil stack joint with vibrational amplitude ranging from 15 to 25 µm at a clamping pressure of 1.0 bar and a welding time of 0.6 s. Amplitude of 15 µm caused relatively limited vibration motion leading to visible gap. As amplitude increased, visible gap was minimized, but cracks and tearing appeared from 25 µm. It was confirmed that a reduction in the effective thickness of the foil stack appeared around the indentation with surface cracks when the welding energy exceeded 900 J, due to excessive welding energy causing severe plastic deformation. This optimal energy threshold and the degradation above 900 J are consistent with previous multilayer welding studies [17,18,19,20].

Table 5 shows the cross-sectional view of foil stack joint with clamping pressure ranging from 0.5 to 1.5 bar at a vibrational amplitude of 20 µm and a welding time of 0.6 s.

It was found that indentation on anvil side significantly increased at 1.5 bar, leading to decreased effective thickness. Accordingly, it revealed that variation in clamping pressure had more sensitive effect on indentation size and depth of joint among welding parameters.

To establish a unified interpretation across different parameter sweeps, the joint-profile observations in Section 3.1 are next re-plotted against welding energy. This approach collapses the effects of welding time, vibrational amplitude, and clamping pressure onto a single process-integrated axis, enabling (i) quantitative scaling of indentation geometry and (ii) an energy-based quality window for the 60-layer foil stack.

### 3.2. Effect of Welding Energy on Length and Depth of Indentations

**Figure 2:**
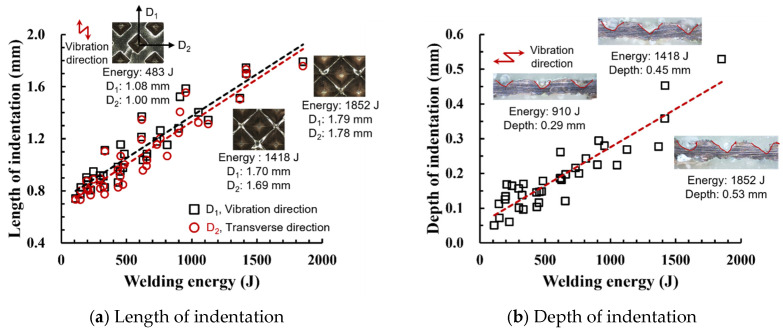
Indentation growth behavior of foil stack joints with increasing welding energy (Dotted lines indicate linear trendlines drawn as guides to the eye to emphasize the monotonic increase with welding energy).

Figure 2 shows the indentation growth behavior of foil stack joints with increasing welding energy. The dotted lines in Figure 2 are linear trendlines provided as guides to the eye to highlight the overall scaling tendency of indentation evolution with welding energy. As shown in Figure 2a, the indentation length D1 (parallel to the vibration direction) and the transverse indentation width D2 were measured over welding energies from 110 J to 1852 J. Both D1 and D2 increase monotonically with welding energy and exhibit an approximately linear dependence. Notably, D1 is consistently comparable to or slightly larger than D2, suggesting preferential indentation elongation along the vibration direction under oscillatory shear.

To characterize the indentation profile in the thickness direction, the indentation depth was measured at three locations (I1, I2, and I3) across the knurled contact region, and the average depth was evaluated over the same energy range (Figure 2b). Specifically, the indentation depth was measured at three positions along the vibration direction: I1 near the front fileted edge of the sonotrode horn (rounded with R2.5), I2 at the central region of the knurled contact, and I3 near the rear fileted edge (rounded with R2.0). The indentation depth increases approximately linearly with welding energy, while the local depth follows the order I2 > I3 > I1. This non-uniform depth distribution is attributed to geometric and loading asymmetry of the horn/knurl region (e.g., local height differences associated with filet machining at the front and rear sides of the sonotrode horn), as well as possible non-uniform normal pressure arising from a bending moment when the normal force is applied from the transducer side region. Overall, Figure 2 demonstrates that higher welding energy leads to progressive indentation growth in both in-plane dimensions (D1, D2) and the thickness direction (depth), reflecting increased local plastic deformation under combined normal pressure and high-frequency oscillatory loading. Similar linear correlations between indentation geometry and welding energy have been reported in ultrasonic welding literature [17,18,19]. The indentation depth directly affects the fracture behavior in multilayer foil joints, as demonstrated by Kang and Lee [19] for ultrasonic-welded copper foils, where excessive indentation led to premature interfacial failure.

### 3.3. Classification of Joint Quality Based on Welding Energy

In ultrasonic welding, joint formation is governed by coupled interfacial frictional heating and severe plastic deformation under oscillatory shear. The machine-reported welding energy integrates the instantaneous power delivered during the weld and is therefore widely used in process monitoring as a practical surrogate descriptor of the net process input. Accordingly, we adopt welding energy as a unifying variable to merge different parameter sets into a single quality map and to define a reproducible process window for the 60-layer Cu foil stack.

As shown in Table 6, the joint profiles of foil stack were characterized into three categories as follows: Insufficient heat input welding—when the welding energy is below 500 J, the density of indentations was the lowest, exhibiting numerous interfacial gaps and insufficient material compaction due to insufficient welding energy. Appropriate heat input welding—when the welding energy ranged from 500 to 900 J, the indentations were properly formed, indicating a penetration depth of approximately 30–40% of the total thickness of the foil stack. Excessive heat input welding—when the welding energy is above 900 J, the density and penetrated depth of the indentations are highest, exhibiting tearing and cracks on the joint surface with reduced effective thickness of foil stack due to excessive plastic deformation. Through the observation of the joint profile, the range of indentation length and depth according to the classification of joint quality was determined. The energy-based failure mode categories are further rationalized in Section 3.4 using SEM/EDS evidence, where interfacial gap/void closure, oxygen-enriched interfacial signatures, and micro-crack formation are directly compared across the three quality classes. The vibrational amplitude has been identified as a dominant parameter influencing weld quality and indentation characteristics in ultrasonic metal welding [20], which aligns with the amplitude-dependent trends observed in the present 60-layer foil stack.

### 3.4. Microstructural Characteristics

**Figure 3:**
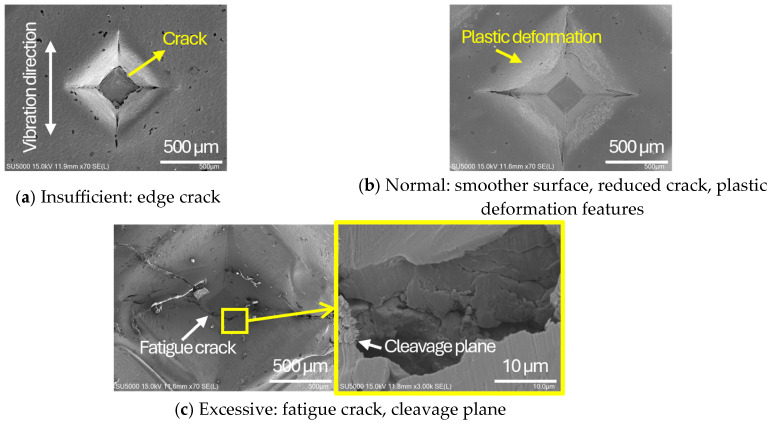
Indentation surface SEM images representing the three energy-based joint quality categories (obtained at fixed clamping pressure of 1.5 bar and vibrational amplitude of 25 µm, with varying welding time/energy): (**a**) insufficient energy (under-welding) condition (0.2 s, 197 J), (**b**) adequate energy (normal-welding) condition (0.6 s, 759 J), and (**c**) excessive energy (over-welding) condition (0.8 s, 1127 J) with a magnified SEM picture of crack propagated area.

**Figure 4:**
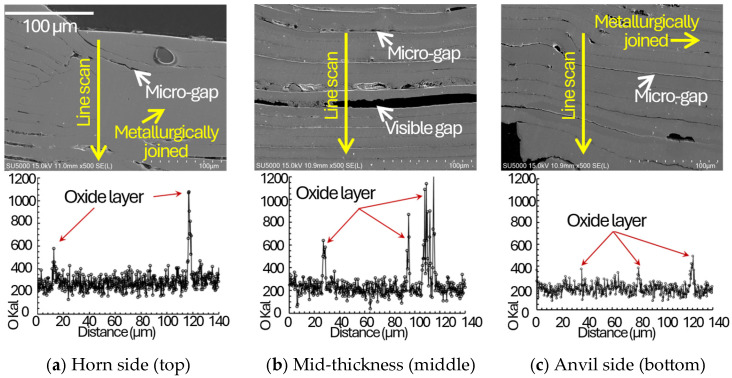
Cross-sectional SEM images with EDS oxygen line scans for the insufficient energy (under-welding) condition at three representative thickness locations: (**a**) horn side region, (**b**) mid-thickness region, and (**c**) anvil side region. Micro gaps/visible gaps remain along the joining interfaces, and pronounced oxygen peaks are detected at the interfaces, consistent with incomplete disruption/removal of surface oxides and insufficient intimate contact.

**Figure 5:**
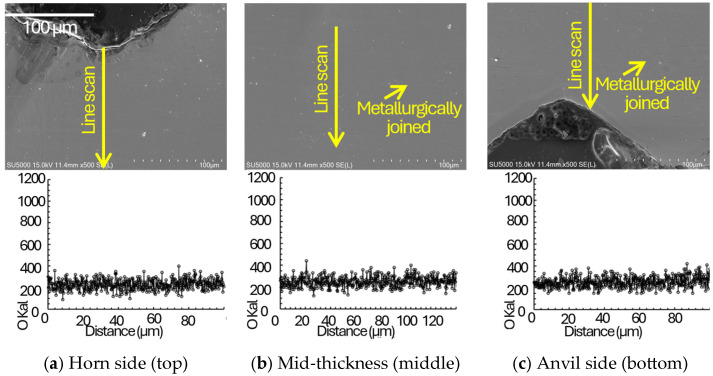
Cross-sectional SEM images with EDS oxygen line scans for the adequate energy (normal-welding) condition at three representative thickness locations: (**a**) horn side region, (**b**) mid-thickness region, and (**c**) anvil side region. Interfacial gaps are largely suppressed and high-intensity oxygen peaks are not pronounced, indicating improved intimate contact and interfacial bonding across the stack thickness.

**Figure 6:**
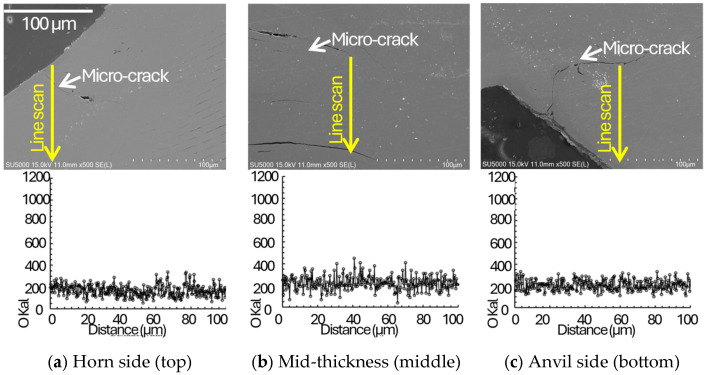
Cross-sectional SEM images with EDS oxygen line scans for the excessive energy (over-welding) condition at three representative thickness locations: (**a**) horn side region, (**b**) mid-thickness region, and (**c**) anvil side region. Although interfacial bonding without pervasive gaps is observed, micro-cracks appear across the thickness, consistent with damage accumulation under excessive plastic deformation and cyclic oscillatory shear.

To mechanistically support the proposed energy-based quality classification, SEM observations were performed on the indentation surface and on polished cross-sections, together with EDS oxygen line scans across representative joining interfaces at the horn side, mid-thickness, and anvil side regions (Figure 3, Figure 4, Figure 5 and Figure 6). In ultrasonic welding, interfacial bonding develops through the coupled action of oscillatory shear and normal pressure, which promotes (i) disruption and expulsion of surface films/contaminants, (ii) local plastic flow that drives closure of interfacial gaps/voids and increases the true contact area, and (iii) frictional heating that enhances interfacial activation at surface asperities. The primary bonding mechanisms occur through mechanical mixing and dynamic recrystallization at the interface [21]. Accordingly, the interfacial gap state (micro/visible gaps versus gap closure) and oxygen-enriched interfacial signatures (EDS O line-scan peaks) are used here as morphology-based indicators of interfacial integrity and bonding continuity. Advanced process monitoring techniques using real-time sensor feedback have been successfully applied to detect such interfacial integrity variations during ultrasonic metal welding [21,22], enabling in-line quality assessment without destructive testing.

The indentation surface SEM images (Figure 3) show distinct damage signatures across the three categories. Under insufficient energy, crack initiation near the indentation edge indicates localized stress concentration associated with knurl penetration under cyclic shear. Within the adequate energy window, the smoother indentation appearance with reduced cracking is consistent with enhanced local plastic flow and progressive closure of near-surface interfacial gaps around the indentation. Under excessive energy, fatigue-related cracking on the indentation surface indicates damage accumulation under repeated high-frequency oscillatory loading.

In Figure 4, Figure 5 and Figure 6, cross-sectional images of the joints taken by SEM and corresponding EDS are plotted. For the insufficient energy condition (Figure 4), persistent micro/visible gaps together with pronounced oxygen-enriched interfacial signatures suggest incomplete disruption of oxide-related interfacial features and insufficient true contact, which limits the formation of a continuous bonded interface. Non-uniform energy distribution through multilayer stacks has been observed in previous studies [22]. Under insufficient energy, interfacial gaps were more frequently observed in the stack interior (mid-thickness) than near the tool-adjacent regions, suggesting delayed interfacial closure toward the interior as welding energy increases. In the adequate energy window (Figure 5), interfacial gaps/voids are largely closed and oxygen-enriched interfacial signatures are not pronounced across the thickness, indicating that film disruption/expulsion and gap closure have progressed sufficiently to establish higher bonding continuity through the stack. Systematic optimization approaches have been successfully applied to multilayer battery tab welding [23,24]. For the excessive energy condition (Figure 6), bonding is still observed without pervasive gaps; however, micro-cracks appear across the thickness, implying that excessive plastic deformation and the associated strain hardening, combined with cyclic oscillatory shear, promote crack initiation and growth. Excessive indentation and degradation at high energy levels are common over-welding phenomena [20,21]. Therefore, the adequate energy window can be interpreted as a balance between achieving closure of interfacial gaps/voids (improved contact state and bonding continuity) and avoiding damage accumulation (micro-cracking) characteristic of over-welding. Although ultrasonic welding can impose severe plastic deformation and may locally modify the near-interface microstructure (e.g., subgrain formation or grain refinement), the present study focuses on interfacial integrity indicators that directly govern peel-dominated failure in ultra-thin Cu foils. In particular, interfacial gap/void closure, oxygen-enriched interfacial signatures (EDS O line-scan peaks), and micro-crack formation provide a direct microstructural/morphological basis for the observed transitions in failure modes. High-resolution characterization (e.g., EBSD or TEM) would be valuable to quantify grain-scale refinement and dislocation substructures and is therefore suggested as future work.

### 3.5. Mechanical Properties

**Figure 7:**
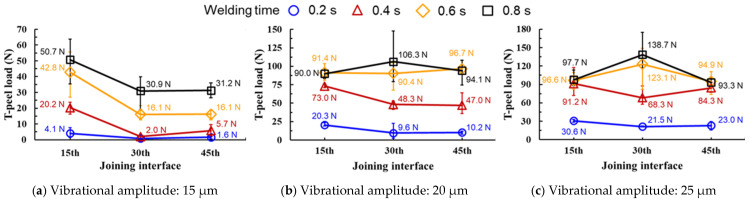
Interface-resolved T-peel peak load as a function of welding time at a fixed clamping pressure of 1.5 bar: (**a**) 15 µm, (**b**) 20 µm, and (**c**) 25 µm vibration amplitude. The datasets correspond to the 15th, 30th, and 45th joining interfaces within the 60-layer stack.

Figure 7 presents interface-resolved T-peel peak loads for three vibration amplitudes. In each panel, welding time is the independent variable (x-axis), while the joining interface position (15th, 30th, and 45th) is reported as separate datasets to reveal how the optimum welding time depends on the interface depth within the 60-layer stack. Clamping pressure is fixed at 1.5 bar in all panels to isolate the effects of welding time (within each panel) and vibration amplitude (between panels). Figure 7a shows a comparison of failure load with different welding times at a clamping pressure of 1.5 bar and a vibrational amplitude of 15 µm. The maximum failure load of 50.7 N was obtained at the 15th joining interface under a welding time of 0.8 s. Interface-dependent strength variations similar to those observed here have been reported for multilayer ultrasonic welds [22,23,25]. Shin and de Leon [18] reported comparable parametric effects in similar ultrasonic spot welding of aluminum alloy sheets, where welding time and amplitude significantly influenced joint strength.

It can be seen that even with insufficient vibrational amplitude, the required welding energy increases. However, the increase in value in failure load at the 30th and 45th joining interfaces is relatively decreased due to the limited vibrational motion. Figure 7b shows a comparison of failure load with different welding times at a clamping pressure of 1.5 bar and a vibrational amplitude of 20 µm. The maximum failure load of 106.3 N was obtained at the 30th joining interface under a welding time of 0.8 s. Furthermore, it can be observed that the failure load at the 15th and 45th joining interfaces decreased at a welding time of 0.8 s compared to the welding time of 0.6 s. This result indicated that excessive welding energy caused cracks and a reduction in effective thickness in the material near the horn and anvil of the foil stack joint due to excessive plastic deformation as the welding time increased. On the other hand, the failure load at the 30th joining interface increases proportionally with the increase in vibrational amplitude and welding time, which is thought to be due to material compaction effects and strain hardening.

Figure 7c shows a comparison of failure load with different welding times at a clamping pressure of 1.5 bar and a vibrational amplitude of 25 µm. The maximum failure load of 138.7 N was obtained at the 30th joining interface under a welding time of 0.8 s. Interface-dependent strength variations similar to those observed here have been reported for multilayer ultrasonic welds [23,25]. The T-peel test configuration is particularly sensitive to interfacial bonding quality in dissimilar metal joints, as demonstrated by Ye et al. [25] for Cu-Al ultrasonic welds, where failure modes transitioned from interfacial separation to base metal tearing with increasing welding energy.

Particularly, it was confirmed that the failure load at the 15th joining interface was higher at a welding time of 0.4 s compared to 0.6 and 0.8 s, when considering the deviation values. This suggests that the joint on the horn side was excessively damaged at that welding time of 0.6 and 0.8 s. Moreover, a relatively uniform nugget formation along the thickness direction of the foil stack is observed at a welding time of 0.2 s.

**Figure 8:**
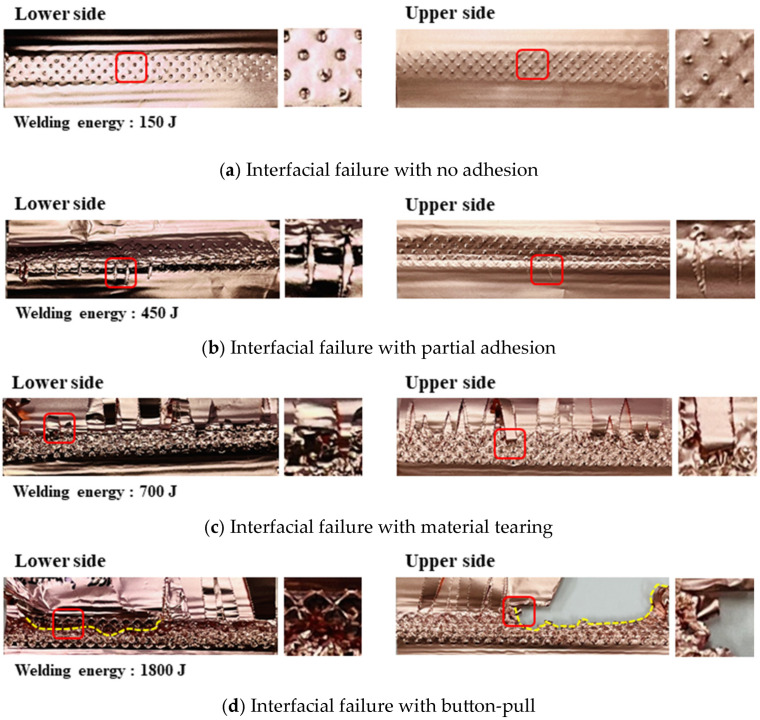
Representative failure modes at the 30th joining interface as a function of welding energy (mid-thickness interface selected for representativeness); Magnified pictures at the right side are from the red rectangular area in the left side picture.

Figure 8 summarizes the failure mode transition at the 30th joining interface, which represents the mid-thickness region where the highest and most stable T-peel loads were obtained. The same energy-dependent transition trend was also observed at the 15th and 45th interfaces, but with different critical energies due to the proximity to the horn/anvil and the associated stress/strain localization.

Figure 8a shows that the interfacial failure with no adhesion was observed in the range of welding energy from 0 to 300 J. The interfacial detachment at the joining interface was clearly visible and the indentation of sonotrode tip was shallow due to insufficient welding energy being transmitted to joining interface. This failure mode indicates low peel load (below 5.0 N) due to no adhesion of the material. Figure 8b shows that the interfacial failure with partial adhesion was observed in the range of welding energy from 300 to 500 J. The minimal adhesion in the joining area existed locally at the indentation to be contacted with sonotrode tip. It signifies the initial stage in the development of the joining interface. Moreover, this failure mode indicates insufficient heat input welding conditions. Figure 8c shows that the interfacial failure with material tearing was observed in the range of welding energy from 500 to 900 J. The failure surface leaves much parent material attached at the boundary of indentation with material tear. It can be inferred that as the welding energy increases a sufficient joining has been developed; this failure mode indicates appropriate heat input welding conditions. These failure mode transitions are typical indicators of progressive bond quality in ultrasonic welding [22,23,25]. Ni et al. [23] systematically characterized failure mode transitions in ultrasonic-welded copper joints and correlated them with microstructural evolution at the bonding interface, supporting the energy-based classification approach adopted in the present study.

Figure 8d shows that the interfacial failure with button-pull was observed in the range of welding energy from 900 to 1800 J. The failure surface exhibited that through-thickness tearing (nugget pull-out fracture) occurred at the nugget surface due to excessive plastic deformation and the presence of cracks on the indentation surface. Meanwhile, the tearing failure occurred in the remaining surface. This failure mode indicates excessive heat input welding conditions. These failure mode transitions are typical indicators of progressive bond quality in ultrasonic welding [23,25].

**Figure 9:**
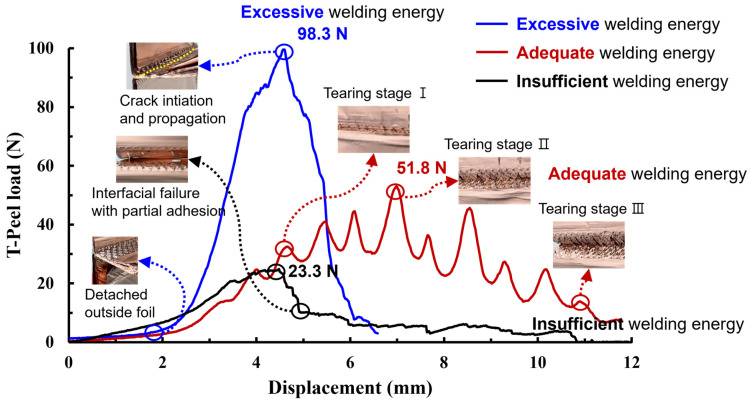
Validation of the energy-based quality classification: representative load–displacement curves and corresponding fracture appearances for insufficient, adequate, and excessive energy conditions at the 30th joining interface.

Figure 9 provides an independent validation of the energy-based quality classification by showing that each energy category corresponds to a distinct load–displacement signature and fracture morphology at the 30th joining interface. The insufficient heat input welding was characterized by interfacial separation with no adhesion or partial adhesion. It was observed that the T-peel load gradually increases until the point of failure, and then it significantly decreases, converging to zero or resulting in tearing in part of the adhered material. In the case of appropriate heat input welding, material tearing was observed overall, with the remaining attached part on the joint after the T-peel test. Moreover, it can be seen that the welded joint sequentially fractures depending on the peeling direction, exhibiting multiple failure points during the T-peel test. Excessive heat input welding over welding was characterized by button-pull failure, with partial fractures occurring around the perimeter of joint surface. When the maximum failure load was the highest, the outside foil detached from the joint when the T-peel load was below approximately 5 N due to the presence of tearing and material thinning caused by excessive plastic deformation. After that, cracks initiate and propagate from the indentation, which leads to a significant decrease in load after the peak point of the T-peel load.

Compared with published ultrasonic welding studies on lower-layer foil stacks or foil-to-tab configurations, the present 60-layer foil stack joint exhibits a distinct interface dependence, where the mid-thickness interface (30th) provides the most stable and highest T-peel peak load within the adequate energy window. Amplitude and clamping pressure have been identified as dominant parameters affecting Cu-Cu ultrasonic weld quality [20,21,22]. Because “welding energy” is machine-reported and system-dependent, direct numerical comparison of absolute energy across different equipment is not strictly equivalent; therefore, we benchmark our results primarily using comparable geometric/mechanical indicators (e.g., indentation fraction of total thickness and peak-load ranges) while emphasizing the process-window concept for high-layer-count stacks.

### 3.6. Implications for Electrical Resistance in Battery Applications

Although electrical resistance/conductivity was not directly measured in the present study, the morphological indicators used for the energy-based quality classification are directly relevant to the expected electrical performance of Cu foil stack joints in battery current paths. In multilayer Cu joints, the joint resistance is often governed not only by the bulk resistivity of copper but also by contact-related contributions (e.g., constriction resistance and interfacial film/oxide effects), which depend strongly on the real contact area and the continuity of the bonded interfaces. Therefore, interfacial gap/void closure and the suppression of oxygen-enriched interfacial signatures (EDS O line-scan peaks), as observed in Section 3.4, are expected to reduce contact resistance by increasing the effective current-carrying area and mitigating oxide-related barriers at the joining interfaces. Real-time process monitoring can enhance quality control in multilayer ultrasonic welding applications [21,24].

Based on this rationale, the insufficient energy (under-welding) condition, characterized by persistent micro/visible interfacial gaps and oxygen-enriched interfacial signatures, would likely exhibit relatively higher joint resistance due to reduced effective contact area and incomplete disruption of oxide-related interfacial features. In contrast, within the adequate energy window, the closure of interfacial gaps/voids and improved bonding continuity across the thickness would be expected to provide a lower-resistance current path. For the excessive energy (over-welding) condition, interfacial bonding is still present; however, the appearance of micro-cracks and the possibility of localized thickness reduction associated with excessive indentation may introduce current path discontinuities and local increases in resistance, which can be detrimental in battery applications due to potential hotspot formation under high-current operation. Accordingly, while mechanical integrity is the primary focus of this work, the present morphology-based classification provides a physically grounded expectation that the adequate energy window is also favorable from an electrical resistance perspective. Direct four-terminal (Kelvin) resistance measurements across the joined region will be pursued in future work to quantitatively validate these correlations.

## 4. Conclusions

In the present study, the joining of a foil stack composed of 60 layers of 8 µm thick Cu foils was successfully achieved using linear vibration ultrasonic welding process, and the following conclusions were obtained.

1. Based on cross-sectional observations across different energy/time conditions, bonding features were first observed near the tool-contacted outer layers and then extended toward the mid-thickness region as welding energy increased, suggesting a progressive development of bonded regions through the stack thickness.
2. In the multilayered foil stack joint, thickness-dependent SEM/EDS observations suggest a tool proximity effect in bond development: interfaces adjacent to the horn and anvil tend to exhibit earlier gap/void closure, whereas the mid-thickness interfaces require higher energy to achieve comparable interfacial closure and bonding continuity (Section 3.4; Figure 4, Figure 5 and Figure 6). This progression is inferred from the spatial distribution of interfacial gaps/voids and oxygen-enriched interfacial signatures and is consistent with the dynamic material–behavior framework reported in Ref. [3]. Direct time-resolved imaging was not performed in the present study; therefore, the sequence is presented as an evidence-based inference rather than a directly observed temporal evolution. Subsequently, nugget formation at the interface of middle region due to the generation of frictional heat as slip appears with compressive and vibrational loading.
3. The maximum failure load of 138.7 N was obtained at the 30th joining interface under a welding time of 0.8 s at a clamping pressure of 1.5 bar and a vibrational amplitude of 25 µm. For the horn side interface (15th), the peak load did not monotonically increase with welding time at higher amplitudes, and the apparent optimum shifted when considering the scatter, indicating early damage sensitivity near the tool-contacted region under excessive energy. This implies that the joint on the horn side was excessively damaged at that welding time of 0.6 and 0.8 s.
4. In terms of their failure modes and load–displacement curves obtained from T-peel test, in the case of appropriate heat input welding, material tearing was observed overall, with the remaining attached part on the joint after the T-peel test. Moreover, it can be seen that the welded joint sequentially fractures depending on the peeling direction, exhibiting multiple failure points during the T-peel test.

## Figures and Tables

**Table 1:** Chemical composition and mechanical properties of material used [2].

**Chemical Composition (wt. %)**			
Cu	O		Other
>99.99	<0.0005		Bal.
**Mechanical Properties (After 10 min at 130 °C)**			
Tensile strength (MPa)		Elongation (%)	
326		9.7	

**Table 2:** Ultrasonic welding conditions for Cu foil stack.

Input Value	Setting Range
Clamping pressure (bar)	0.5, 1.0, 1.5 (3 levels)
Vibrational amplitude (µm)	15, 20, 25 (3 levels)
Welding time (s)	0.2, 0.4, 0.6, 0.8 (4 levels)
Welding energy (J)	110~1852

**Table 3:** Joint profiles of foil stacks in various welding times ranging from 0.2 to 0.8 s; indentation depth values are reported as mean ± SD from five repeated welds for each condition (*n* = 5).

Welding Time and Corresponding Welding Energy	0.2 s (197 J)		0.4 s (456 J)		0.6 s (759 J)		0.8 s (1127 J)	
**Horn side, ** 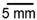	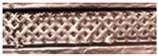		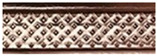		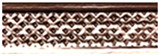		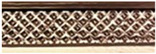	
**Indentation size** **D_1_, D_2_ (mm)**	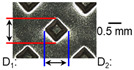		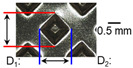		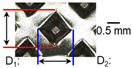		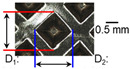	
	0.84	0.82	1.12	1.10	1.26	1.20	1.34	1.31
**Indentation depth (mm)**	0.134 ± 0.013		0.147 ± 0.015		0.200 ± 0.021		0.268 ± 0.03	
**Cross-section**	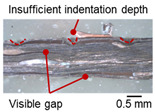		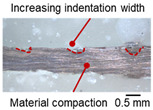		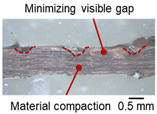		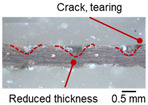	

**Table 4:** Joint profiles of foil stacks in various vibrational amplitudes ranging from 15 to 25 µm; indentation depth values are reported as mean ± SD from five repeated welds for each condition (*n* = 5).

Vibrational Amplitude and Corresponding Welding Energy	15 μm (474 J)		20 μm (759 J)		25 μm (1020 J)	
**Horn side, ** 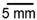	** 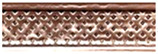 **		** 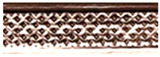 **		** 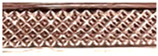 **	
**Indentation size** **D_1_, D_2_ (mm)**	** 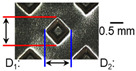 **		** 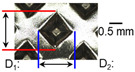 **		** 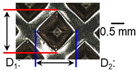 **	
	0.98	0.85	1.26	1.20	1.40	1.32
**Indentation depth (mm)**	0.148 ± 0.004		0.200 ± 0.024		0.224 ± 0.061	
**Cross-section**	** 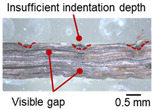 **		** 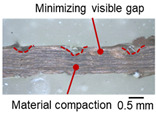 **		** 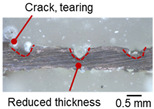 **	

**Table 5:** Joint profiles of foil stacks in various clamping pressures ranging from 0.5 to 1.5 bar; indentation depth values are reported as mean ± SD from five repeated welds for each condition (*n* = 5).

Clamping Pressure and Corresponding Welding Energy	0.5 Bar (452 J)		1.0 Bar (759 J)		1.5 Bar (954 J)	
**Horn side, ** 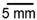	** 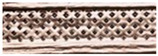 **		** 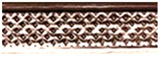 **		** 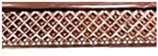 **	
**Indentation size** **D_1_, D_2_ (mm)**	** 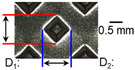 **		** 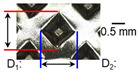 **		** 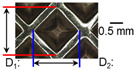 **	
	0.95	0.91	1.26	1.20	1.58	1.55
**Indentation depth (mm)**	0.115 ± 0.003		0.200 ± 0.023		0.279 ± 0.081	
**Cross-section**	** 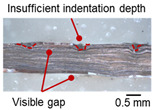 **		** 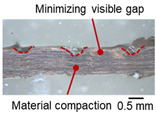 **		** 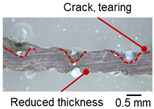 **	

**Table 6:** Energy-based joint quality classification using the machine-reported welding energy (Ew = ∫P(t)dt), supported by the corresponding indentation morphology and cross-sectional integrity.

Classification	Insufficient Welding Energy	Adequate Welding Energy	Excessive Welding Energy
Welding Energy (J)	Below 500	500~900	Above 900
**Appearance**	** 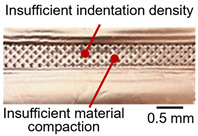 **	** 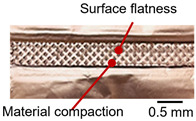 **	** 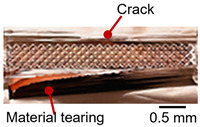 **
**Indentation length (mm)**	0.73~1.10 (D_1_)	1.10~1.28 (D_1_)	1.28~1.79 (D_1_)
**Indentation depth (mm)**	0.05~0.18 (Avg.)	0.18~0.22 (Avg.)	0.22~0.53 (Avg.)

## Data Availability

The original contributions presented in this study are included in the article. Further inquiries can be directed to the corresponding authors.
